# Relationship between Social Skills and Happiness: Differences by Gender

**DOI:** 10.3390/ijerph18157929

**Published:** 2021-07-27

**Authors:** Carlos Salavera, Pablo Usán

**Affiliations:** Faculty odf Education, University of Zaragoza, 50009 Zaragoza, Spain

**Keywords:** social skills, happiness, teaching school (primary education), gender

## Abstract

This study examines the relationship between social skills and happiness in 1st-year Teaching School students, as well as possible gender differences. The sample comprised 243 Teaching School students (Primary Education) in Zaragoza, including 110 men (45.27%) and 133 women (54.73%), aged 18–25 (average age 20.23 years; s.d. = 1.586). In order to analyse the relationship between social skills and subjective happiness, the Scale of Social Skills and Subjective Happiness Scale were used. While men scored higher in all social skills-related factors, women scored higher in all factors related to happiness. The study shows that factors such as self-expression in social settings and the ability to say no and cut off social interactions have a direct and significant effect on happiness among men, while self-expression in social settings and the ability to express anger led to a higher perception of happiness among women. Similarly, situations such as asking for and defending rights have an indirect and significant effect in men, reducing their levels of happiness. In the case of women, no social skills factors were found that led to lower happiness. It may be concluded that significant gender differences exist, although broader and lateral studies are needed in order to examine the relationship between gender identities, social skills and subjective happiness more in depth, and thus, understand the effect of these constructs in the development of personality.

## 1. Introduction

Social skills are acquired mainly through learning (observation, imitation, information, rehearsal…), and include both verbal and non-verbal behaviours. They also suppose effective and appropriate initiatives and responses; they serve to increase social reinforcement [1].

Some people have extraordinary social skills. They can establish competent social relationships with others, and perform a reinforcing role, standing as a model for those with whom they interact.

They easily fit in social settings, seamlessly collecting information during social interactions while gratifying those with whom they interact. These people are generally labelled as extroverted, assertive and socially able. Daily interactions, for instance in the classroom, clearly show that these traits are not universal, and that, in fact, many people struggle with social interaction. The importance of possessing good social skills is increasingly clear, most particularly since the beginning of the pandemic. Relations with others not only allow individuals to find a place in different social groups and contexts, but also play a role in reinforcing their own personality [2,3]. 

Prosocial play skills not only contribute to trigger reinforcing relationships, but also increase the likelihood that the individual will be sought by others in the future [4,5]. On the other hand, it is important to note that children who are less skilful in play and who interact less with their peers will grow to have less social skills. This can lead to irreversible situations of isolation in the future. 

Social skills, or lack thereof, can play a role in the emergence of internalising and externalising problems, crystallising as factors of protection and risk. The absence or inadequate use of these skills can lead to the individual forming wrong views of reality. 

In other words, social skills are deployed in interpersonal contexts to express feelings, attitudes, opinions and demands in a context-appropriate manner, respecting these same conducts in others; this leads to immediate problems being solved and possible future problems being avoided [6,7]. Generally, these skills are acquired through learning, imitation, trial and error, testing and information-gathering. They are learned abilities, rather than innate skills. There are two major types of social skill: basic and complex. The former have to be internalised before the latter can be learned. The process begins in childhood and continues during adolescence, during which communicative and relational tools are developed with the aim to establish positive social relationships, which are sources of self-satisfaction and personal wellbeing [8]. Let us remember that social skills are necessary for effective and satisfactory social interactions, ensuring the gratification of others and avoiding punishment and rejection [9].

In the current social context, having good social skills is especially necessary. Teaching schools must stimulate the use and practice of social abilities, both in terms of teaching practices and mutual social interactions among future teachers. The use of such constructs as self-esteem, emotions, affects and social skills among students must be encouraged by the teaching staff. 

From a teaching perspective, encouraging these traits not only facilitates the integration of the individual in a social setting, but also provides them with a better understanding of their own personal traits. For many of our students, having a good relationship with their peers is a target in itself, as well as an important reinforcing factor from a social and academic point of view [10,11]. 

In this regard, it is worth emphasising that lacking in social skills can have negative consequences in the long term, such as school dropout, poor problem-solving skills and unsatisfactory social relationships [12,13].

On the other hand, subjective happiness provides the individual with a positive outlook for medium-term achievement, but tends to blur long-term prospects [14,15]. Therefore, its time range is limited. Happiness has been defined as subjective well-being, a personal, subjective and global evaluation of the cognitive and emotional state of the individual [16]; happiness improves the individual’s outlook and prevents the tendency to “overthink” negative problems. Some authors have even affirmed that happiness is a better indicator of quality of life than well-being or health [17]. 

Happiness has also been defined as the valuation of life as a whole, posing how issues such as poverty, loneliness and illness make this happiness difficult [18]. On the other hand, there are studies that associate happiness with higher levels of physical, emotional and social well-being [19]. Happier people tend to have stronger immune systems, more stable partners, more creative ideas, even higher income [20]. They tend to be more outgoing, prosocial, and empathetic [21,22]. As such, a happy person will have friends and people they can trust, as well as the resources to achieve their goals and cope with stressful events [23]. Positive emotions can focus on the past, present, and future; emotions related to the future crystallize in hope and trust, those related to the present in calm and fluidity (optimal experience), and those related to the past in self-realization, satisfaction, pride and serenity [24]. In short, happiness is a positive emotion focused on the present, highly desirable for the person, but difficult to achieve. The role of teachers’ happiness in the performance of schoolchildren has been proven in different investigations [25,26], hence the importance of working on the happiness construct with future teachers.

Finally, according to different investigations, social skills have an important impact on happiness, finding how the level of social connections is significantly correlated with the level of happiness [27,28]. 

### Social Skills, Happiness and Gender

In different investigations, women have obtained higher scores on social skills tests than men, report higher levels of interpersonal functioning and show more concern for the quality of their interpersonal relationships [29,30,31]. 

Some studies with university students reported how women obtained better grades in conversational skills with assertive opposition and empathic skills [32]. However, other research works have pointed out gender differences in social skills [28], and have attributed fewer social skills and higher levels of anxiety in social situations to men [33]. It is also worth noting that the gender role has not come as clearly to other researchers, who have indicated that the social context has a stronger effect on these differences in social competence [34]. Some other studies have reported the difficulty of obtaining conclusive results [35].

Regarding how happiness behaves by gender, it cannot be said that the results of different investigations have been conclusive. Some studies [36,37,38] have found that women show higher levels of happiness than men, while others [39,40,41,42,43,44] have found no significant differences in terms of happiness depending on gender. In samples of university students, significant differences by gender have been found. Thus, women perform more behaviours to achieve happiness [45,46,47]. 

According to the previous antecedents, the aim of this study is to analyse the relationship between social skills, subjective happiness and gender among teaching school students (primary school). The two starting hypotheses are as follows: (1) women show higher social skills and happiness than men; (2) the social skills factors involved in happiness are related to gender.

## 2. Materials and Method

### 2.1. Participants

The sample comprises 243 Teaching School students (primary school) from the Education Faculty, University of Zaragoza, including 110 men (45.27%) and 133 women (54.73%). The participants were selected from among students who were in their 1st year of teaching, so it can be said that a convenience sampling was used. The participants were aged 18–25, with an average age of 20.23 years (s.d. = 1.586). Questionnaires were distributed in class in the presence of the principal investigator. The questionnaires were collected individually as the students finished them, and reviewed to check for errors and to ensure that no questions were left unanswered. One inclusion criterion was the ability to read and communicate in perfect Spanish in order to ensure that the questionnaire was perfectly understood and completed. Exclusion criteria included incomplete questionnaires. The study met the ethical guidelines set out by the Declaration of Helsinki and all ethical principles of research with human beings (voluntary participation; all participants gave their informed consent prior to the study; the participants were fully informed of the nature of the study; personal data were strictly protected and treated confidentially; no form of discrimination was applied; participants could opt out of the study at any point). The survey was designed as an ex post facto prospective study [48]; it used gender as the independent variable and happiness and social skills as the dependent variable. After calculating sample representativeness with a 95% level of confidence and a 5% sampling error, the final surveyed sample was obtained, which proved representative of the province of Zaragoza (Spain). 

### 2.2. Protocol

Upon being handed the survey forms, participants were explained the aim of the study, and the importance of answering all items was emphasised. Participants were given 20 min to fill in the questionnaire. Participants were reminded that all information would be handled anonymously and confidentially. The survey was undertaken during the 2018–2019 academic year.

Statistical analysis was undertaken by means of SPSS 26.0 software. Once the normality of the sample and the equality of variances were established, parametric analytic techniques were chosen. Descriptive analyses of each variable according to gender were undertaken. In all cases, the lowest level of significance was adopted, and differences with a value of *p* < 0.05 were regarded as significant. Bilateral testing was undertaken. For two-group hypothesis testing, we used Student’s *t*-distribution. Equations for the prediction of happiness based on social skills variables were undertaken by logistic regression, using the Stepwise Method (forward path), based on Wald’s statistics. 

### 2.3. Instruments 

#### 2.3.1. Social Skills Scale 

The scale [49], designed and elaborated with Spanish populations, comprises 33 items, 28 of which are scaled in reverse in order to detect deficits in assertion and social skills, and five scaled normally, with four possible answers each. Answers are structured as a four-point Likert scale. The higher the score, the greater the respondent’s social skills and assertiveness. The scores are swiftly and reliably assigned with a template. The instrument provides scores for six different dimensions (each dimension’s Cronbach’s alpha is expressed in brackets): self expression in social settings (0.85); defence of respondent’s consumer rights (0.70); expression of anger or disconformity (0.77); ability to say no and cut off undesired interactions (0.79); ability to make petitions (0.80); and ability to initiate positive interactions with the opposite sex (0.86).

#### 2.3.2. Subjective Happiness Scale (SHS) 

This scale [50] presents a molar measure of wellbeing as a global psychological phenomenon. This scale goes beyond the mere sum of positive and negative emotional states and related cognitive states. In the study, the Spanish adaptation of the scale was used [51] Therefore, the scale measures happiness from the perspective of the respondent, in the understanding that, although different variables can be used to describe happiness, each respondent will have their own perspective of what being happy is and are able to establish whether they are happy or not [50]. The scale comprises four items, and the answers are structured as a Likert scale; scores are added together and divided by the total number of items. In this study, the scale showed a high degree of internal consistency (Cronbach’s alpha = 0.84). 

## 3. Results

The results (Table 1) indicate that gender is a significant factor in all factors under analysis: self-expression in social settings; defence of respondent’s consumer rights; expression of anger or disconformity; ability to say no and cut off undesired interactions; ability to make petitions; and ability to initiate positive interactions with the opposite sex. This suggests that men and women have significantly different social skills. Although the existing literature suggests that men are more assertive and women are more empathetic, in our study, men yielded higher scores than women in all dimensions. On the other hand, women yielded higher levels of subjective happiness than men.

In order to assess the relations between variables and test the working hypotheses, correlations between scales were calculated according to gender (Table 2). These results suggested that, in men, happiness is positively correlated with self-expression (r = 0.477 **); the ability to express anger (r = 0.373 **); the ability to say no and cut off interactions (r = 0.520 **); and the ability to initiate positive interactions with the opposite sex (r = 0.259 **). Women presented positive correlations between happiness and self-expression (r = 0.270 **); the ability to defend one’s own rights (r = 0.161 **); the ability to express anger (r = 0.273 **); the ability to say no and cut off interactions (r = 0.173 **); and the ability to initiate positive relationships with the opposite sex (r = 0.127 **). That is, self-expression and the ability to say no and cut off interactions have the greatest impact on male self-perception of happiness, and self-expression and the ability to express anger are the weightiest factors among women.

Finally, in order to assess the predicting value of social skills regarding happiness, a large number of hierarchical regressions were undertaken, selecting the factorial scores related to social skills as predictor variables and happiness as a criterion variable. Table 3 shows the steps followed by each model in the prediction of subjective happiness. The variables with the most predicting value among men were the ability to say no and cut off social interactions, self-expression and the ability to make petitions. Among women, the variables with the most predicting value were self-expression and the ability to express anger. Predictor variables explained 68.8% of the variance of the dependent variable (*R*^2^ = 0.688) in the logistic regression model used with men, and 35.5% in that used with women (*R*^2^ = 0.355).

## 4. Discussion

The present study aimed to analyse the relationship between social skills and happiness in first-year Teaching School students (primary education), and examine whether these variables were gender related.

The results suggest that gender differences exist; men yielded higher scores in all variables concerned with social skills, and women higher scores in happiness-related variables. The higher scores yielded by men in social skills could be related to the nature of the sample (recruited among university students), the specific degree they are studying, social factors or teaching by parents of non-traditional gender roles [1,52,53]; in any case, more research is required. The higher degree of subjective happiness shown by women agrees with existing studies [1,54,55]. 

Finally, the predictive value of social skills over happiness was also estimated. The results suggest that four social skills (the ability the say no and cut off interactions; the defence of one’s own rights; self-expression in social settings, and the ability to make petitions) explain the variance to a large extent. Among men, self-expression and the ability to say no were shown to be particularly good predictors of happiness, being negatively correlated with it. Among women, social skills were detected to have a significant predictive value over happiness (especially self-expression and the ability to express anger, which are positively correlated with happiness), although to a lesser extent than among men. This result may be due to the fact that the factor that measures self-expression in social situations aims to measure the ability to express oneself spontaneously and without difficulty in different social situations and when a high score is obtained, the ease of the person to interact, express their feelings and opinions and ask questions in different contexts is revealed, as well as questions related to happiness. Likewise, the expression of anger or disagreement allows the person to give an account of the ability to avoid conflicts or confrontations with other people. This is a surprising result, which requires and encourages further investigation, since existing studies so far suggested that men score higher in social skills related to assertiveness, while women score higher in terms of empathy [56,57,58]. 

In any case, the results of this study suggest that more research is needed. In itself, this is an interesting outcome. The review of the existing literature yields contradictory results, which only emphasises the complex nature of the issue. Some authors argue that women score higher in terms of social skills [59,60,61], but others suggest that correlations between social behaviours are stronger among men [62]. In this regard, it would be desirable to carry out similar studies among students of other degrees as well as outside the student population in order to rule out sample-dependent bias. It would also be advisable to undertake lateral studies in order to determine the acquisition, development and evolution of gender role-related social skills. Vantieghem et al. [53] suggest that gender identities can be associated with certain classroom behaviours, espousing the adoption of gender identity in these studies. The impact of gender identities on school behaviour and academic performance is still imperfectly understood, and more research is needed to analyse this relationship. 

This study presents various limitations: although the size of the sample is acceptable, lateral studies are necessary to assess the evolution of social skills over time. Similar studies must be carried out in other faculties and degrees. Furthermore, the study must be expanded to incorporate other constructs, such as affects, the use of sense of humour and personality traits, which could be strongly related to social skills. Finally, it is possible to point out the possible effects of collinearity between the independent variables, a question that can be resolved in future investigations with the use of clusters as an alternative to multiple regression.

We must emphasise the need to incorporate training in social skills to the school curricula of future schoolteachers, especially given its direct link to primary education and its direct relationship with subjective happiness. The results of this study encourage us to formulate other questions and define methodologies with which to improve the socio-affective development of teachers. 

These methodologies can provide future school teachers with tools for their everyday tasks, allowing them to do their jobs more competently, which will have positive repercussions on their subjective happiness. Sometimes, social skills are perceived as something complementary or accessory to technical skills, and that social skills are thus a minor factor when it comes to teaching. Teachers hide behind technical abilities to not become fully involved in the work of their students. In fact, social skills are generally assumed, not only at work, but also in their private lives, but this is often not entirely justified. Teaching schools must encourage the use of social tools to contribute to the overall performance of teaching professionals.

## 5. Conclusions

In conclusion, this study lends substantial empirical support to the model that states that social skills are a necessary trait for teaching professionals, and that they can contribute to the subjective happiness not only of teachers, but of students too. The study also suggests that significant gender differences exist in terms of social skills and subjective happiness. Finally, the results emphasise the need to encourage the use of social skills to promote participation in academic, social and school activities that contribute to a good professional performance and to the subjective happiness of both teachers and students.

## Figures and Tables

**Table 1:** Social ability scores and happiness by gender.

	Males		Females			
	Mean	s.d.	Mean	s.d.	*t*	Sig.
Self expression in social settings	26.34	3.52	24.11	4.02	5.386	0.000
Defence of respondent’s consumer rights	15.61	1.91	12.93	2.42	10.867	0.000
Expression of anger or disconformity	12.05	2.14	11.36	2.14	3.045	0.002
Say no and cut off undesired interactions	17.22	3.09	15.34	2.95	6.002	0.000
Make petitions	17.89	2.21	16.26	2.50	6.311	0.000
Initiate positive interactions with the opposite sex	16.12	1.76	14.24	2.14	8.613	0.000
Happiness	19.76	4.02	21.28	4.20	−3.484	0.001

**Table 2:** Correlations between social skills and happiness by gender.

	Self Expression in Social Settings	Defence of Respondent’s Consumer Rights	Expression of Anger or Disconformity	Say No and Cut Off Undesired Interactions	Make Petitions	Initiate Positive Interactions with the Opposite Sex
Males	0.477 **	0.129	0.373 **	0.520 **	−0.025	0.259 **
Females	0.270 **	0.161 **	0.273 **	0.173 **	−0.019	0.127 **

**: Correlation is significant at the level 0.01.

**Table 3:** Social skills as predicting factors of happiness according to gender.

	Variable	*B*	*e.t.*	*R* ^2^	*t*	Sig
Males	(constant)	16.051	1.160		13.832	0.000
	Say no and cut off undesired interactions	2.824	0.454	0.520	6.219	0.000
	Defence of respondent’s consumer rights	−2.327	0.426	0.593	−5.457	0.000
	Self expression in social settings	1.349	0.334	0.647	4.037	0.000
	Make petitions	−1.119	0.338	0.688	−3.313	0.001
Females	(constant)	16.118	0.658		24.486	0.000
	Self expression in social settings	0.831	0.174	0.273	4.766	0.000
	Expression of anger or disconformity	0.967	0.198	0.335	4.893	0.000

Note. *B*: Beta Coefficient; *e.t.*: estimated error; *R*^2^: R squared; *t*: value of t; Sig: Significance. Excluded variables: Interaction with the opposite sex.

## Data Availability

The data presented in this study are available on request from the corresponding author.
